# Seasonal fluctuation of total water intake and hydration status among young men and women: a prospective cohort study

**DOI:** 10.3389/fnut.2024.1463501

**Published:** 2025-01-30

**Authors:** Jianfen Zhang, Na Zhang, Junbo Lu, Shufang Liu, Yongwei Lin, Guansheng Ma

**Affiliations:** ^1^Department of Nutrition and Food Hygiene, School of Public Health, Peking University, Beijing, China; ^2^National Institute for Nutrition and Health, Chinese Center for Disease Control and Prevention, Beijing, China; ^3^Laboratory of Toxicological Research and Risk Assessment for Food Safety, Peking University, Beijing, China; ^4^National Center for Occupational Safety and Health, Beijing, China; ^5^School of Public Health, Hebei University Health Science Center, Baoding, China

**Keywords:** seasonal fluctuations, total water intake, hydration status, males, females

## Abstract

**Background:**

Water intake and hydration status have been reported to fluctuate throughout the year. This study investigated seasonal fluctuations of total water intake and hydration status among young adults in Baoding, China.

**Methods:**

This prospective cohort study enrolled 82 young adults aged 18–23 years in Baoding, China. Total drinking fluids consumed and water from food were assessed, and the osmolality and electrolyte concentrations of 24-h urine and fasting blood samples were determined. Differences among the four seasons were compared by mixed linear models, followed by determinations of least-significant differences (LSD), with spring used as the reference.

**Results:**

Seventy-nine participants (43 men and 36 women) completed the study. Total water intake (TWI) was 359 ~ 429 mL higher in spring and summer than in autumn and winter and was 116 mL higher in summer than in winter (all *p* < 0.05). Chinese recommendations for TWI were met by 13.9% to 22.8% of participants, and recommendations for total fluid intake were met by 10.1% to 16.5%, but these differences were not statistically significant (*p* > 0.05). Urinary and plasma biomarkers differed significantly among the four seasons (*p* < 0.05), with osmolality and urine specific gravity (USG) being significantly higher in summer than in other seasons (*p* < 0.05). The percentage of participants with optimal hydration status increased from 38.0% in summer to 62.0% in spring (*p <* 0.05). Men had more concentrated urine as well as higher plasma osmolality and solute concentrations than women during each season of the year (*p <* 0.05).

**Conclusion:**

TWI and urinary and plasma biomarkers of hydration were found to vary seasonally among Chinese young adults, with hydration status being poorer in summer. Men need to pay more attention than women to maintain optimal hydration status.

**Clinical trial registration:**

https://www.chictr.org.cn/showproj.html?proj=124857, ChiCTR2100045268.

## 1 Introduction

Adequate water intake is necessary to maintain optimal hydration status and wellbeing. Dehydration, the process of losing water, has been found to impair endurance performances (1–3) and even the cognitive performance, including short-term memory and attention, among pilots, athletes, young adults, children, and the elderly (4–8). In addition, chronic dehydration was shown to be a risk factor for urinary calculi, bladder cancer, obesity, or other diseases (9–11). Overhydration is also detrimental to health, as it can lead to hyponatremia. In contrast, optimal hydration status, or euhydration, has been associated with lower rates of urinary tract infections, hypertension, and fatal coronary heart diseases.

Hydration status reflects the balance between water intake and loss. Water requirements vary by gender, age, and environment. Intake of fluids may also vary during different seasons of the year, affecting hydration status. Biomarkers for evaluating hydration status include a loss of body weight and urinary factors, including urine volume, osmolality, color, and urine specific gravity (USG) (12–14). Moreover, plasma, saliva, and tear osmolality were also found to be sensitive to changes in hydration status (15). No single standard method is currently available to determine hydration status in all environments, resulting in the condition-dependent application of biomarkers. A healthy sedentary adult is expected to lose a moderate amount of water, ranging from 1,800 mL to 3,000 mL, in an environment maintained at 18–25°C (16), with each 1°C increase in temperature increasing fluid intake by 5.69 g/d (17). Environmental conditions, including temperature and humidity, have been reported to affect the amount of water intake, but those studies did not evaluate hydration status (17–20). Because hydration status has been positively associated with water intake (21–23), changes in season may alter hydration status.

Studies in China have estimated that hydration status in spring and winter was suboptimal in approximately ~25.0–37.1% of participants, but those studies did not compare hydration status in spring and winter (24, 25). Despite the importance of preventing dehydration, data on hydration status in different seasons are limited, particularly in free-living conditions. Most research in this area has assessed the effects of heat exposure in summer on heat strain and cardiovascular response, with hydration status also examined. One study showed that workers were dehydrated at the start and end of their work day in summer, with 70% of these workers having heat stress had USG ≥1.020 (26, 27).

Studies investigating the effects of seasonal variations on hydration status have yielded contradictory results. For example, season did not affect hydration status, as assessed by urine indices, in children (20, 28–30), whereas elderly people in Japan were more dehydrated in spring than in summer (31). USG in children has been reported higher in winter than in summer (32), whereas loss of body weight in young adults was greater in summer than in winter (33). These contradictory findings were likely due to differences in the indices evaluating hydration status, the methods of assessing the impact of different seasons, and the age group of the participants.

These results suggested that young adults would have higher total water intake (TWI) and higher total drinking fluid intake, but more dehydrated, in summer than in the other seasons of the year. This study therefore examined total drinking fluid intake and hydration biomarkers in a sample of young adults in free-living conditions to estimate the effects of season on hydration status. These findings may enable the determination of whether higher temperature and humidity lead to poorer hydration status.

## 2 Materials and methods

### 2.1 Participants

This prospective cohort study enrolled young adults living in Baoding, Hebei Province, China. Recruitment notices were sent through WeChat and QQ (Tencent Holdings Ltd., Shenzhen, China), instant messaging apps widely used by college students. In addition, a campus talk on recruitment open to all college students was held. The study inclusion and exclusion criteria have been reported (34). Healthy males and females aged 18–23 years were included in the study. However, participants who were smokers, habitually consumed alcohol (>20 g/day) high levels of caffeine (>250 mg/day), and those who had chronic or other diseases were excluded from the study. Finally, the study recruited a total of 82 young adults, 44 men and 38 women.

### 2.2 Sample size calculation

Related studies among young male and female adults reported that the standard deviation of total drinking fluids was 468 mL (24, 34). Calculations using SAS software (SAS Institute Inc., Cary, NC) showed that, with *δ* set at 158 mL, *α* set at 0.05, and a drop-out rate of 20%, 74 participants were needed.

### 2.3 Study procedure

**Figure 1:**
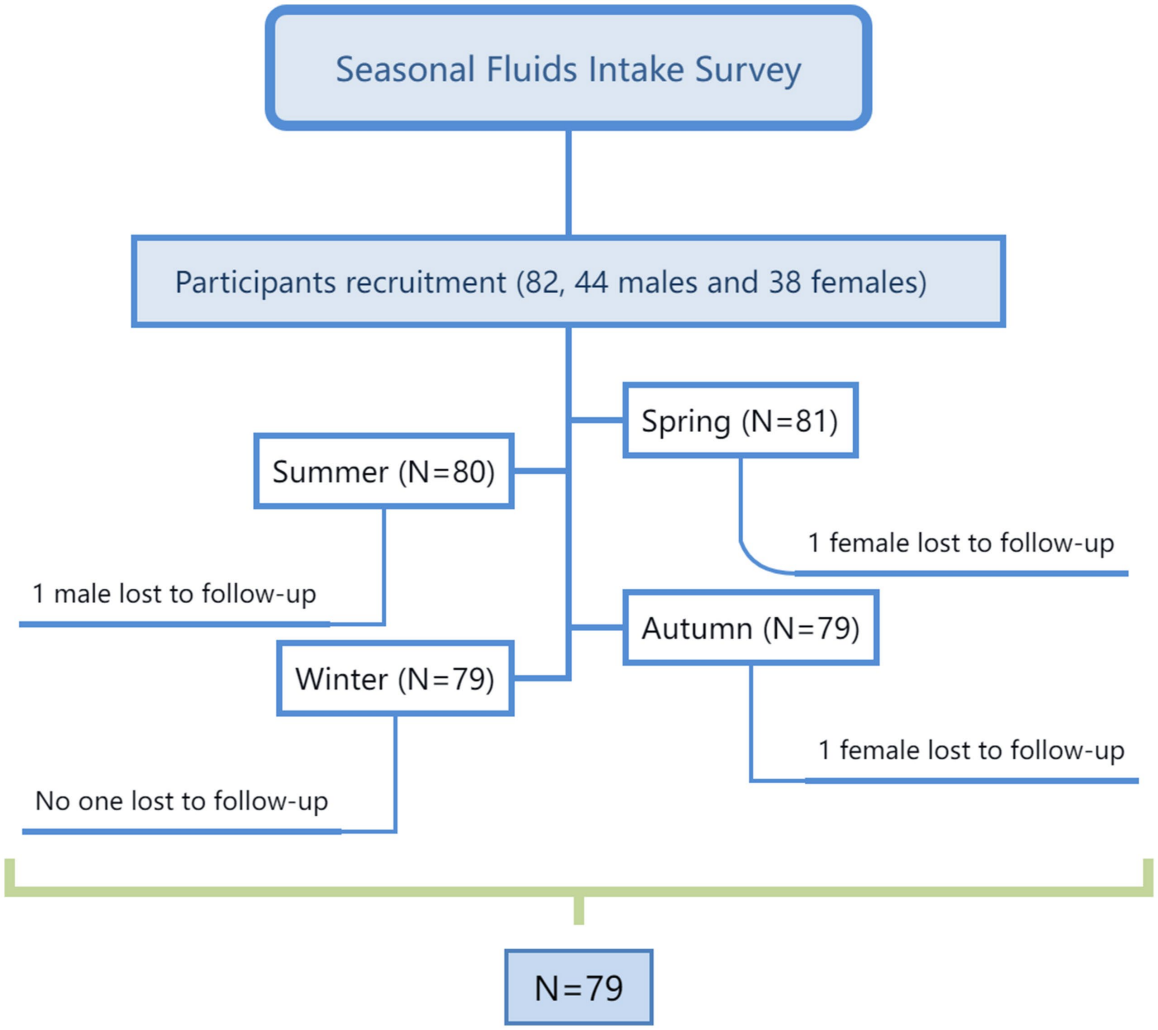
The flow chart.

**Figure 2:**
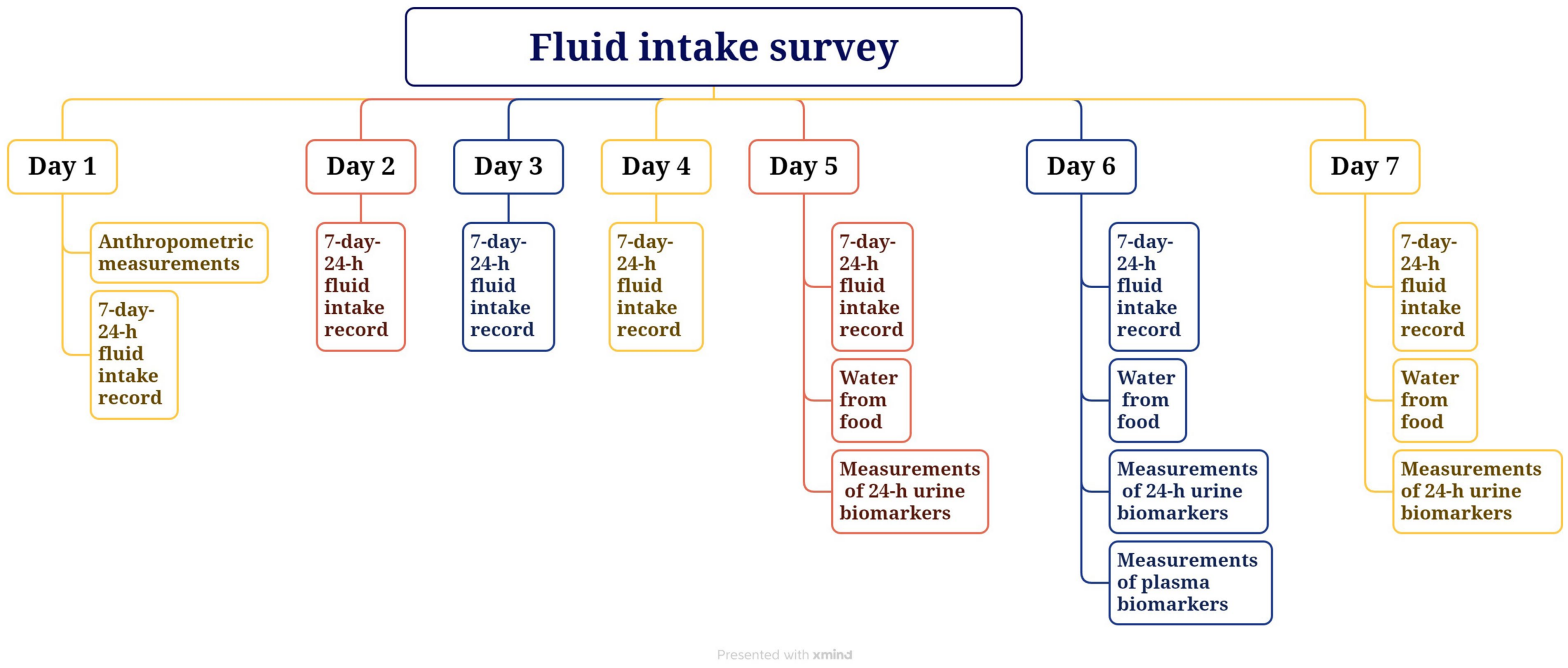
The study procedure.

The study was carried out over 7 consecutive days each, 5 weekdays and 2 weekend days, in April, June, October, and December 2021, or a total of 28 days. On the first day of each study period, anthropometric measurements, including height, weight, and waist circumference, were taken. All participants were instructed to use the self-designed 7-day 24-h fluid intake questionnaire, with high validity and reliability (35, 36), to record the intake of fluids. In addition, all foods eaten for 3 consecutive days (2 weekdays and 1 weekend day) during each 7-day period were weighed and recorded. Participants collected 24-h urine samples, including the first morning urine, on days 5 to 7 (2 weekdays and 1 weekend day) of each 7-day period, and fasting blood samples of all participants were collected on day 6. Indoor and outdoor temperature and humidity were recorded each day using the WSB-1-H2 (Exasace, Zhengzhou, China). The study flow chart is shown in Figure 1. The study procedure is shown in Figure 2.

#### 2.3.1 Anthropometric measurements

Height was measured two times to the nearest 0.1 cm, and weight was measured two times to the nearest 0.1 kg; waist circumference was assessed two times the nearest 0.1 cm with the participants wearing light clothes and no footwear (HDM-300; Huaju, Zhejiang, China; Accu Measure, Greenwood Village, CO, United States). Body mass index (BMI) was calculated as weight (kg)/height squared (m).

#### 2.3.2 Measurement of total fluid intake

Total fluid intake was determined using a self-designed 7-day 24-h fluid intake record questionnaire. Participants were asked to drink any fluids using the cups that were accurate to the nearest 5 ml, and they were asked to record the type and amount of drinking fluid each time, which had been described in our previous study (37). In order to ensure the compliance of the participants, we established group leaders to supervise participants to record details of fluids intake; furthermore, we asked them to submit the questionnaire of previous day on the next morning and we checked the time, amounts, and related information of the total fluids intake; finally, we compared the amounts of the total fluids intake and the volume of urine according to the questionnaire. Water from food was measured using a duplicate portion method, with samples of food being collected and sent to the laboratory immediately (37).

### 2.4 Hydration biomarkers

Urinary biomarkers of hydration status included urine volume, osmolality, pH, USG, and electrolyte concentrations in 24-h urine samples. These samples, including the second urine of the first day and the first morning urine of the second day, were collected by participants using containers provided by the researchers, which were immediately sent to the laboratory and stored at +4°C. There were several methods to maintain the compliance of the urine collection. The participants were asked to immediately send every urine sample to the laboratory after collection using a black opaque bag. All of the participants were college students, and they were instructed to maintain their normal free - living conditions. The laboratory was on the campus, which was in the middle of the teaching building, dormitory building, and school cafeteria. During daytime hours, portable refrigerators were carried by our investigators to areas where participants frequently gathered. Subsequently, the investigators took charge of sending the urine samples to the laboratory. Or, participants could choose to send their urine samples to the laboratory on their own. We would still be in the laboratory from 5:00 am to 11:30 pm, so the participants could send urine samples themselves to the laboratory during this time. If they were not convenient to deliver, they could give the urine sample to the investigators in each building, then the investigators would send them to the laboratory. At night, each floor of the dormitory building was equipped with a portable refrigerator and so that the participants could put the urine samples into the refrigerator at night, then the investigators would send them to the laboratory in the morning.

Urine volume was measured to the nearest 0.1 kg using a desktop electronic scale (YP20001, SPC, Shanghai, China); osmolality was assessed with freezing point method (SMC 30C; Tianhe, Tianjin, China); USG, pH were evaluated by automatic urinary sediment analyzer with uric dry-chemistry method (H-800; Dirui, Changchun, China) and the electrolyte concentrations were determined by automatic biochemical analyzer with the ion-selective electrode potentiometer method (including sodium, potassium, chloride, calcium, magnesium and phosphate; AU 5800; Beckman, Brea, CA, United States) were tested by experienced laboratory technicians using standard procedures, as previously described (37).

Plasma biomarkers of hydration status included osmolality, electrolyte concentrations (including sodium, potassium, chloride, calcium, magnesium, and phosphate), and creatinine and blood urea nitrogen (BUN) concentrations. All of these biomarkers were tested in fasted blood obtained on day 6 by experienced laboratory technicians using standard procedures (37).

### 2.5 Statistics

Data were presented as mean ± standard deviation (SD) or number (%). Optimal hydration was defined as urine osmolality ≤500 mOsm/kg and dehydration as urine osmolality >800 mOsm/kg (24, 38). Differences during the four seasons of the year were compared using mixed linear models, with adjustments for sex, age, and BMI, followed by determinations of least-significant differences (LSD), with spring set as the reference. All statistical analyses were performed using SAS 9.2 software (SAS Institute Inc., Cary, NC, United States), with statistical significance set at *p* < 0.05.

## 3 Results

### 3.1 Study participants

Of the 82 subjects recruited, 79 (96.3%), consisting of 43 men and 36 women, completed the study. Table 1 shows the characteristics of the participants during all four seasons of the year. Mean weight and BMI were found to differ significantly during the four seasons (*p* < 0.05).

**Table 1:** Demographic characteristics of the study subjects by seasons of the year.

	Spring			Summer			Autumn			Winter		
	Males (*n* = 43)	Females (*n* = 36)	Total (*n* = 79)	Males (*n* = 43)	Females (*n* = 36)	Total (*n* = 79)	Males (*n* = 43)	Females (*n* = 36)	Total (*n* = 79)	Males (*n* = 43)	Females (*n* = 36)	Total (*n* = 79)
Age (y)	19.9 ± 0.5	19.9 ± 0.5	19.9 ± 0.5	19.9 ± 0.5	19.9 ± 0.5	19.9 ± 0.5	19.9 ± 0.5	19.9 ± 0.5	19.9 ± 0.5	19.9 ± 0.5	19.9 ± 0.5	19.9 ± 0.5
Height (cm)	174.7 ± 5.6	163.0 ± 5.9	169.4 ± 8.2^c^	175.1 ± 5.6	162.7 ± 5.9	169.4 ± 8.4^c^	174.8 ± 5.7	164.2 ± 6.2	170.0 ± 7.9^abd^	174.2 ± 5.5	163.9 ± 6.1	169.5 ± 7.7^c^
Weight (kg)	72.4 ± 15.2	57.9 ± 12.3	65.8 ± 15.7^bcd^	71.7 ± 14.4	57.3 ± 12.5	65.1 ± 15.3^acd^	74.3 ± 14.8	58.9 ± 13.1	67.2 ± 16.0^ab^	74.5 ± 14.7	59.1 ± 13.5	67.5 ± 16.1^ab^
BMI (kg/m^2^)	23.6 ± 4.5	21.8 ± 4.4	22.8 ± 4.5^bcd^	23.3 ± 4.3	21.6 ± 4.4	22.6 ± 4.4^acd^	24.3 ± 4.5	21.8 ± 4.6	23.1 ± 4.7^abd^	24.5 ± 4.4	22.0 ± 4.8	23.4 ± 4.8^abd^

Data presented as mean ± standard deviation or *n* (%). BMI, body mass index. ^a^There are statistically significant differences compared with spring; ^b^there are statistically significant differences compared with summer; ^c^there are statistically significant differences compared with autumn; ^d^there are statistically significant differences compared with winter.

### 3.2 Temperature and humidity

Average indoor temperatures for the 7 days in spring, summer, autumn, and winter were 23.4°C, 27.0°C, 23.0°C, and 22.6°C, respectively, whereas average outdoor temperatures were 18.1°C, 28.1°C, 14.7°C, and 2.7°C, respectively (Table 2). Average indoor humidity during these four seasons was 48, 47, 44, and 40%, whereas average outdoor humidity was 55, 47, 52, and 63%, respectively. Indoor and outdoor temperatures differed significantly during the four seasons (*p* < 0.05), whereas indoor and outdoor humidity did not (*p* > 0.05).

**Table 2:** Temperature and humidity over 7 days during each of the four seasons.

	Indoors		Outdoors	
	Temperature (°C)	Humidity (%)	Temperature (°C)	Humidity (%)
Spring	23.4 ± 0.5^†‡^	48 ± 5	18.1 ± 1.8^†‡^	55 ± 22
Summer	27.0 ± 1.6^##§^	47 ± 10	28.1 ± 2.4^##§^	47 ± 22
Autumn	23.0 ± 0.6	44 ± 6	14.7 ± 1.7^ψ^	52 ± 10
Winter	22.6 ± 0.3^#^	40 ± 4	2.7 ± 2.1^#^	63 ± 16

Data presented as mean ± standard deviation or *n* (%). ^†^significant differences between spring and summer; ^‡^significant differences between spring and autumn; ^#^significant differences between spring and winter; ^##^significant differences between summer and autumn; ^§^significant differences between summer and winter; ^ψ^significant differences between autumn and winter.

### 3.3 Total water intake

TWI, total fluid intake, and water from food differed significantly during the four seasons of the year (all *p* < 0.05). TWI was 359 ~ 429 mL higher in spring and summer than in autumn and winter and 116 mL higher in summer than in winter (Table 3). Total fluid intake did not differ significantly in autumn, spring, and winter (*p* > 0.05). Chinese recommendations for TWI were met by 13.9 to 22.8% of participants, with the highest percentage in spring, but these differences were not statistically significant (*p* > 0.05). Chinese recommendations for total fluid intake were met by 10.1 to 16.5% of subjects, with the highest percentage in summer, but these differences were also not statistically significant (*p* > 0.05).

**Table 3:** Characteristics of total water intake among young adults.

	Spring			Summer			Autumn			Winter		
	Male (*n* = 43)	Female (*n* = 36)	Total (*n* = 79)	Male (*n* = 43)	Female (*n* = 36)	Total (*n* = 79)	Male (*n* = 43)	Female (*n* = 36)	Total (*n* = 79)	Male (*n* = 43)	Female (*n* = 36)	Total (*n* = 79)
Total water intake (mL)	2,856 ± 692	2,169 ± 546^*^	2,543 ± 714^**^	2,772 ± 726	2,116 ± 421	2,473 ± 687	2,552 ± 566	1939 ± 442	2,273 ± 596^‡^	2,332 ± 628	1856 ± 491	2,115 ± 614^#^
Total water intake (mL/kg/d)	39.2 ± 8.2	38.6 ± 11.4^*^	39.0 ± 9.8^**^	38.5 ± 7.3	38.6 ± 10.0	38.6 ± 8.6^##§^	35.1 ± 9.1	34.3 ± 10.2	34.7 ± 9.5^‡^	31.8 ± 8.1	32.5 ± 10.2	32.1 ± 9.1^#^
Percentage meeting adequate total water intake (based on Chinese recommendations) (%)	12 (27.9)	6 (16.7)	18 (22.8)	11 (25.6)	4 (11.1)	15 (19.0)	9 (20.9)	3 (8.3)	12 (15.2)	7 (16.3)	4 (11.1)	11 (13.9)
Percentage meeting the total water intake recommended by the EFSA (%)	32 (74.4)	20 (55.6)	52 (65.8) ^**^	29 (67.4)	20 (55.6)	49 (62.0)^##§^	22 (51.2)	15 (41.7)	37 (46.8)^‡^	16 (37.2)	10 (27.8)	26 (32.9)^#^
Total fluid intake (mL)	1,398 ± 612	924 ± 458^*^	1,182 ± 594 ^**†^	1,408 ± 525	992 ± 365	1,216 ± 502^##§^	1,221 ± 494	916 ± 398	1,082 ± 475	1,101 ± 402	886 ± 356	1,003 ± 394
Percentage meeting adequate total water intake (based on Chinese recommendations) (%)	6 (14.0)	5 (13.9)	11 (13.9)	8 (18.6)	5 (13.9)	13 (16.5)	6 (14.0)	3 (8.3)	9 (11.4)	6 (14.0)	2 (5.6)	8 (10.1)
Water from food (mL)	1,458 ± 267	1,245 ± 256^*^	1,361 ± 281^**†^	1,364 ± 400	1,124 ± 229	1,255 ± 353^##§^	1,331 ± 273	1,023 ± 209	1,190 ± 289^‡^	1,230 ± 418	970 ± 282	1,112 ± 383^#^

Data presented as mean ± standard deviation or *n* (%). *Significant differences between men and women during the same season; ^**^significant differences among the four seasons; ^†^significant differences between spring and summer; ^‡^significant differences between spring and autumn; ^#^significant differences between spring and winter; ^##^significant differences between summer and autumn; ^§^significant differences between summer and winter; ^ψ^significant differences between autumn and winter.

### 3.4 Hydration biomarkers

Table 4 shows mean urinary volume; osmolality; Na, K, Cl, Ca, phosphate, and Mg concentrations; USG and pH of the 24-h urine samples. Urine samples from men were more concentrated than those from women during each season (*p* < 0.05), with the proportions of women having optimal hydration status being higher than those of men during each of the four seasons (all *p* < 0.05).

**Table 4:** Properties of 24-h urine samples obtained from young adults.

	Spring			Summer			Autumn			Winter		
	Male (*n* = 43)	Female (*n* = 36)	Total (*n* = 79)	Male (*n* = 43)	Female (*n* = 36)	Total (*n* = 79)	Male (*n* = 43)	Female (*n* = 36)	Total (*n* = 79)	Male (*n* = 43)	Female (*n* = 36)	Total (*n* = 79)
24-h volume (mL)	1,205 ± 361	1,273 ± 505	1,235 ± 431^†‡^	1,068 ± 378	1,083 ± 452	1,075 ± 411^##§^	1,331 ± 455	1,328 ± 505	1,329 ± 475^ψ^	1,207 ± 453	1,125 ± 504	1,170 ± 475
24-h urinary osmolality (mOsm/kg)	531 ± 179	429 ± 129^*^	484 ± 165^†^	662 ± 178	533 ± 206^*^	603 ± 201^##^	498 ± 148	420 ± 142^*^	462 ± 149^ψ^	629 ± 194	555 ± 213	595 ± 205^#^
Numbers of subjects with osmolality ≤500 mOsm/kg, *n* (%)	49 (62.0%)^†^			24 (30.4%)			44 (55.7%)^ψ^			27 (34.2%)^#^		
Void	5.1 ± 1.2	4.9 ± 1.0	5.0 ± 1.1^‡^	5.0 ± 1.3	4.7 ± 1.0	4.9 ± 1.2^##^	5.6 ± 1.3	5.1 ± 1.1	5.3 ± 1.3^ψ^	5.1 ± 1.3	4.5 ± 1.1	4.8 ± 1.2^*^
Na (mmol/L)	144 ± 44	126 ± 39	136 ± 43^†^	173 ± 53	144 ± 57^*^	160 ± 56^##§^	144 ± 42	127 ± 43	136 ± 43^ψ^	149 ± 45	143 ± 48	134 ± 41^#^
K (mmol/L)	25.42 ± 10.34	27.84 ± 10.51	26.5 ± 10.4	31.48 ± 10.55	32.44 ± 13.35	31.9 ± 11.8	27.47 ± 9.72	27.60 ± 10.05	27.5 ± 9.8^ψ^	27.14 ± 9.80	30.01 ± 11.44	28.5 ± 10.6^#^
Cl (mmol/L)	135 ± 43	118 ± 38	127 ± 42^†^	165 ± 49	137 ± 54^*^	152 ± 53^##§^	132 ± 38	115 ± 37	124 ± 38^ψ^	137 ± 41	131 ± 41	134 ± 41^#^
Ca (mmol/L)	2.83 ± 1.19	2.36 ± 0.94	2.62 ± 1.10^‡^	2.86 ± 1.15	2.39 ± 1.30	2.64 ± 1.23^##^	2.00 ± 0.79	1.78 ± 0.82	1.90 ± 0.81^ψ^	2.62 ± 1.08	2.31 ± 1.04	2.48 ± 1.07
Phosphate (mmol/L)	13.05 ± 5.79	10.38 ± 4.04^*^	11.83 ± 5.22^†^	19.06 ± 7.22	15.20 ± 6.98^*^	17.30 ± 7.32^##^	15.18 ± 7.46	11.69 ± 4.94^*^	13.60 ± 6.63^ψ^	17.15 ± 8.48	15.32 ± 6.44	16.31 ± 7.62^#^
Mg (mmol/L)	3.01 ± 1.18	2.83 ± 1.04	2.93 ± 1.12^‡^	3.08 ± 1.20	2.74 ± 1.08	2.93 ± 1.15^##^	2.41 ± 1.12	2.34 ± 0.98	2.38 ± 1.05^ψ^	2.84 ± 1.09	2.89 ± 1.12	2.86 ± 1.10
USG	1.017 ± 0.005	1.014 ± 0.004^*^	1.015 ± 0.005^†‡^	1.019 ± 0.006	1.016 ± 0.006^*^	1.018 ± 0.006^##§^	1.016 ± 0.005	1.012 ± 0.004^*^	1.014 ± 0.005^ψ^	1.017 ± 0.005	1.015 ± 0.005	1.016 ± 0.005^#^
USG ≥ 1.020	5 (12.8%)			5 (12.8%)			5 (12.8%)			5 (12.8%)		
pH	6.8 ± 0.4	7.0 ± 0.3^*^	6.9 ± 0.4^†‡^	6.6 ± 0.4	6.8 ± 0.4	6.7 ± 0.4^##§^	6.9 ± 0.4	7.1 ± 0.3^*^	7.0 ± 0.3^ψ^	6.9 ± 0.3	6.9 ± 0.4	6.9 ± 0.3

Data presented as mean ± standard deviation or *n* (%). *Significant differences between men and women during the same season; ^**^significant differences among the four seasons; ^†^significant differences between spring and summer; ^‡^significant differences between spring and autumn; ^#^significant differences between spring and winter; ^##^significant differences between summer and autumn; ^§^significant differences between summer and winter; ^ψ^significant differences between autumn and winter.

Significant differences observed in urinary volume (*F* = 12.089, *p* < 0.001), osmolality (*F* = 42.109, *p* < 0.001), void (*F* = 7.762, *p* < 0.001), Na (*F* = 317.166, *p* < 0.001), K (*F* = 581.290, *p* < 0.001), Cl (*F* = 18.466, *p* < 0.001), Ca (*F* = 23.268, *p* < 0.001), P (*F* = 25.841, *p* < 0.001), Mg (*F* = 10.101, *p* < 0.001), USG (*F* = 28.857, *p* < 0.001), and pH (*F* = 16.561, *p* < 0.001).

Urine volumes were significantly higher in summer, winter, and spring than in autumn (*p* < 0.05), but did not differ significantly in spring and winter (*p* > 0.05). Urine osmolality and USG paralleled urine volume, being highest in summer and lowest in autumn (*p* < 0.05), Moreover, 17.7% ~ 24.0% more participants were in optimal hydration status in spring, autumn, and winter than in summer (*p* < 0.05). Electrolyte concentrations also showed seasonal differences (*p* < 0.05), with Na concentrations being highest in summer and lowest in winter (*p* < 0.05); K concentrations being higher in spring, summer, and autumn than in winter (*p* < 0.05); Cl concentrations being highest in summer and lowest in spring and autumn (*p* < 0.05); Ca and Mg concentrations being highest in summer and lowest in autumn (*p* < 0.05); phosphate concentrations being higher in summer and winter than in spring (*p* < 0.05); and pH being lowest in winter (*p* < 0.05).

### 3.5 Plasma indices

Mean plasma hydration biomarkers, including osmolality and concentrations of Na, K, Cl, Ca, phosphate Mg, creatinine, and blood urea nitrogen (BUN) in fasting blood samples, are presented in Table 5. All of these biomarkers were within their normal physiological ranges. Plasma hydration biomarkers were found to differ significantly in men and women during spring, summer, autumn, and winter (*p* < 0.05).

**Table 5:** Composition of blood samples obtained from young adults.

	Spring			Summer			Autumn			Winter		
	Male (*n* = 43)	Female (*n* = 36)	Total (*n* = 79)	Male (*n* = 43)	Female (*n* = 36)	Total (*n* = 79)	Male (*n* = 43)	Female (*n* = 36)	Total (*n* = 79)	Male (*n* = 43)	Female (*n* = 36)	Total (*n* = 79)
Osmolality (mOsm/kg)	273 ± 6	271 ± 6	272 ± 6^†‡^	291 ± 3	289 ± 5^*^	290 ± 4^##§^	281 ± 2	279 ± 3^*^	280 ± 2^ψ^	285 ± 3	283 ± 5	284 ± 4^*#^
Glucose (mmol/L)	5.4 ± 0.5	5.3 ± 0.4	5.4 ± 0.4	5.4 ± 0.5	5.3 ± 0.5	5.3 ± 0.5	5.5 ± 0.4	5.2 ± 0.5	5.3 ± 0.5	5.2 ± 0.5	5.3 ± 0.5	5.3 ± 0.5
Na (mmol/L)	142 ± 1	140 ± 1^*^	141 ± 1^†^	141 ± 1	140 ± 1^*^	140 ± 1	141 ± 4	140 ± 1	140 ± 3	141 ± 1	141 ± 1	141 ± 1
K (mmol/L)	3.78 ± 0.30	3.86 ± 0.27	3.8 ± 0.3^†‡^	3.94 ± 0.26	3.98 ± 0.28	4.0 ± 0.3^##§^	4.73 ± 0.40	4.62 ± 0.29	4.7 ± 0.4^ψ^	4.19 ± 0.25	4.15 ± 0.26	4.2 ± 0.3^#^
Cl (mmol/L)	104 ± 2	105 ± 1^*^	105 ± 1^†^	103 ± 1	105 ± 1^*^	104 ± 2^##§^	103 ± 3	104 ± 1^*^	103 ± 1	102 ± 1	104 ± 1	103 ± 1^*#^
Ca (mmol/L)	2.43 ± 0.05	2.42 ± 0.06	2.42 ± 0.06^†^	2.42 ± 0.07	2.38 ± 0.07^*^	2.40 ± 0.07^§^	2.44 ± 0.06	2.42 ± 0.07	2.43 ± 0.06^ψ^	2.49 ± 0.06	2.46 ± 0.07	2.47 ± 0.07^#^
Phosphate (mmol/L)	1.24 ± 0.14	1.19 ± 0.11	1.22 ± 0.13^†‡^	1.45 ± 0.16	1.39 ± 0.13	1.42 ± 0.15^##^	1.36 ± 0.14	1.37 ± 0.11	1.37 ± 0.13^ψ^	1.41 ± 0.16	1.45 ± 0.12	1.43 ± 0.14^#^
Mg (mmol/L)	0.87 ± 0.05	0.85 ± 0.05^*^	0.86 ± 0.05^‡^	0.88 ± 0.05	0.85 ± 0.07^*^	0.86 ± 0.06^##§^	0.90 ± 0.05	0.87 ± 0.06^*^	0.89 ± 0.06^ψ^	0.90 ± 0.06	0.88 ± 0.06	0.89 ± 0.06^#^
Creatinine (mmol/L)	69.5 ± 8.7	57.4 ± 8.4^*^	64.0 ± 10.4^†‡^	75.7 ± 6.4	60.4 ± 9.0^*^	68.7 ± 10.8	77.6 ± 7.5	60.6 ± 8.1^*^	69.9 ± 11.5	74.7 ± 6.5	57.9 ± 8.3	67.1 ± 11.2^*#^
Bun (mmol/L)	4.14 ± 0.95	3.81 ± 0.99	3.99 ± 0.97^†^	4.54 ± 1.01	4.11 ± 1.01	4.34 ± 1.02^##^	4.10 ± 0.74	3.57 ± 0.69^*^	3.86 ± 0.76^ψ^	4.50 ± 0.73	4.13 ± 0.75	4.33 ± 0.76^*^

Data presented as mean ± standard deviation or *n* (%). ^*^Significant differences between men and women during the same season; ^**^significant differences among the four seasons; ^†^significant differences between spring and summer; ^‡^significant differences between spring and autumn; ^#^ significant differences between spring and winter; ^##^significant differences between summer and autumn; ^§^significant differences between summer and winter; ^ψ^significant differences between autumn and winter. BUN, blood urea nitrogen.

Significant differences were observed in blood osmolality (*F* = 320.095, *p* < 0.001), Na (*F* = 3.498, *p* = 0.016), K (*F* = 171.036, *p* < 0.001), Cl (*F* = 18.278, *p* < 0.001), Ca (*F* = 26.382, *p* < 0.001), phosphate (*F* = 55.272, *p* < 0.001), Mg (*F* = 10.599, *p* < 0.001), BUN (*F* = 7.846, *p* < 0.001), and Cre (*F* = 16.685, *p* < 0.001).

All plasma biomarkers differed significantly during the four seasons of the year (*p* < 0.05). Osmolality was highest in summer and lowest in spring (*p* < 0.05); Na concentration was lower in summer than during the other seasons (*p* < 0.05); K concentration was highest in autumn and lowest in spring (*p* < 0.05); Cl concentration was higher in spring and summer than in autumn and winter (*p* < 0.05); Ca concentration was lowest in summer and highest in winter (*p* < 0.05); phosphate concentration was highest in winter and lowest in spring (*p* < 0.05); Mg concentration was higher in autumn and winter than in spring and summer (*p* < 0.05); creatinine concentration was higher in summer and winter than in spring and autumn (*p* < 0.05); and BUN concentration was lower in spring than during the other seasons (*p* < 0.05).

## 4 Discussion

To our knowledge, this study is the first to evaluate the effects of season on total drinking fluids intake and hydration biomarkers among young adults in China, finding that urinary osmolality, USG, and electrolyte concentrations were higher in summer than in the other seasons of the year. Moreover, plasma osmolality and the concentrations of some ions were higher in summer, despite TWI and total fluid intake also being higher in summer.

TWI was found to be 359 ~ 429 mL higher in spring and summer than in autumn and winter and 116 mL higher in summer than in winter. A study in Germany also found that TWI increased in summer and decreased in winter (39), and a study of 59 male foresters in Poland reported that TWI was lower in winter (1,009 mL) than in summer (1,189 mL), but similar in winter and autumn (871 mL) (40). Seasonal variations in TWI were likely due to variations in temperature and humidity. The present study also found that the proportions of participants who met Chinese recommendations for TWI and total fluid intake were higher in spring and summer, but the differences were not statistically significant. These findings indicate that fluid intake was stable among young adults, suggesting the need for considerable efforts to change behavior. Moreover, stable fluid intake among adults may indicate a lack of knowledge of recommended fluid intake.

The present study also found that the volume of urine was lowest in summer and highest in autumn, with no significant differences between spring and winter, findings consistent with previous results. For example, a cross-sectional study of young adults showed that the urinary volume was 252.3 mL higher in winter than in summer (41), with warmer temperatures in the summer being associated with reduced urine volume (42). In contrast, other studies found that urine volume did not differ across seasons (43, 44).

This study found that the percentage of participants with optimal hydration status was lowest in summer, accompanied by higher levels of urine osmolality and USG, similar to previous findings (45). These results suggested that participants were more likely to be dehydrated in summer. The similar urine osmolality in summer and winter in the present study may be due to the use of heating equipment in winter 2021 in Baoding, Hebei Province. During that winter, every classroom and dorm room in Baoding was heated, with room temperature maintained at approximately 23°C, possibly increasing water loss from the skin. In addition, young adults spent much more time indoors (20 h) than outdoors (4 h) (19). Although vasopressin concentrations also varied seasonally, peaking during the winter (46), additional studies are required to determine the causes of increased urine osmolality during the winter.

Physiologically, dehydration occurs through passive exposure to a hot environment, exercise, or water restriction. Living in hot environments increased the dehydration rate among free-living subjects. Moreover, 37.2% of Chinese subjects living in a mild climate, with a temperature of 20.7°C, were found to be hydrated (37). Studies assessing seasonal hydration status have reported contradictory results. For example, hydration status, as evaluated using urine biomarkers, did not vary seasonally. USG in summer and winter did not differ significantly among children living in a Mediterranean climate (28) and did not differ seasonally in 60 healthy men (29). Moreover, studies assessing hydration status among adolescents of a mean age of 15.1 years (32) and of elderly subjects in Japan (31) found that USG was higher in winter than in summer. The participants in the present study were aged 18–23 years, the climate of Baoding included four distinctive seasons and hydration biomarkers were evaluated using 24-h urine samples. In contrast, Cyprus has mild winters and hot summers, the participants included adolescents and persons aged >60 years, and hydration markers were evaluated using first morning urine samples. Biomarker concentrations in first morning urine samples were proportional to those in 24-h urine samples, but the former were not as sensitive in monitoring changes in hydration status (22, 23).

Supplemental water intake may be insufficient to maintain hydration status under conditions of high sweat loss, as in summer. The present study found that urine concentrations were higher in summer than during other seasons, whereas an evaluation of 40 male subjects found that the levels of urinary Na and Ca excretion were significantly higher in winter than in summer (47). Na and K excretion levels in the first morning urine samples, however, did not differ seasonally in 104 healthy preschool children (44). Many factors may contribute to these contradictory results, including the age and race of subjects, the climate in which they live, and laboratory testing methods. Measurements of urine osmolality during the four seasons of the year in the present study indicated that approximately 38% ~ 69.6% of participants were either dehydrated or had middle hydration status, depending on the season. Thus, maintaining optimal hydration status is a challenge, even in cold climates.

The results of this study indicated that the prevalence of increased plasma osmolality was higher in summer than in the other seasons, as were plasma concentrations of electrolytes and creatinine. Plasma osmolality was maintained between 275 and 295 mOsm/kg by thirst and vasopressin release. Because of their vital importance in the cardiovascular system, these plasma variables may not be influenced until large amounts of body fluids are lost (47). Indeed, previous studies showed that plasma osmolality remained stable over a wide range of intake of fluids, from 681 mL to 1,135 mL (37, 48). Plasma osmolality ≥290 mOsm/kg is considered indicative of dehydration (49–51). In the current study, plasma osmolality ≥290 mOsm/kg was observed only during the summer, suggesting that participants were at high risk of dehydration during the summer. Moreover, other studies confirmed the seasonal variations of plasma osmolality (31, 52).

The strengths of this prospective cohort study include the participation of young adults in free-living conditions, the collection of 24-h urine samples for 3 consecutive days (2 weekdays and 1 weekend day), and the analyses of these samples by professional investigators.

One limitation of the present study was the subject population, consisting of elite young men and women, suggesting that these findings may not be generalized to other age groups. Furthermore, the sweating of the participants and the total body water measured from the BIA or other methods during each season were not assessed either. Additional studies are needed should examine whether our findings can be confidently extrapolated beyond the participants used.

## 5 Conclusion

TWI, total fluid intake, and hydration biomarkers in urine and plasma were found to vary seasonally among young adults, with TWI and total fluid intake being higher and hydration status lower during the summer than during other seasons. Young adults should be educated on optimal fluid intake, and their hydration status should be monitored during each season of the year.

## Data Availability

The original contributions presented in the study are included in the article/supplementary material, further inquiries can be directed to the corresponding authors.

## References

[1] James LJ Funnell MP James RM Mears SA. Does hypohydration really impair endurance performance? Methodological considerations for interpreting hydration research. Sports Med. (2019) 49:103–14. doi: 10.1007/s40279-019-01188-5, PMID: 31696453 PMC6901416

[2] Francisco R Jesus F Santos P Trbovšek P Moreira AS Nunes C. Does acute dehydration affect the neuromuscular function in healthy adults?–a systematic review. Appl Physiol Nutr Metab. (2024) 49:1441–60. doi: 10.1139/apnm-2024-0192, PMID: 39047298

[3] Francisco R Jesus F Nunes CL Santos P Alvim M Campa F. H2OAthletes study protocol: effects of hydration changes on neuromuscular function in athletes. Br J Nutr. (2024) 131:1579–90. doi: 10.1017/S0007114524000308, PMID: 38299306

[4] Mantantzis K Drewelies J Duezel S Steinhagen-Thiessen E Demuth I Wagner GG. Dehydration predicts longitudinal decline in cognitive functioning and well-being among older adults. Psychol Aging. (2020) 35:517–28. doi: 10.1037/pag0000471, PMID: 32352804

[5] Zhang N Du SM Zhang JF Ma GS. Effects of dehydration and rehydration on cognitive performance and mood among male college students in Cangzhou, China: a self-controlled trial. Int J Environ Res Public Health. (2019) 16:1891. doi: 10.3390/ijerph16111891, PMID: 31146326 PMC6603652

[6] Westfall DR Logan NE Khan NA Hillman CH. Cognitive assessments in hydration research involving children: methods and considerations. Ann Nutr Metab. (2019) 74:19–24. doi: 10.1159/000500341, PMID: 31203295

[7] Goulet EDB Mélançon MO Lafrenière D Paquin J Maltais M Morais JA. Impact of mild hypohydration on muscle endurance, power, and strength in healthy, active older men. J Strength Cond Res. (2018) 32:3405–15. doi: 10.1519/JSC.0000000000001857 28234715

[8] Trangmar SJ González-Alonso J. Heat, hydration and the human brain, heart and skeletal muscles. Sports Med. (2019) 49:69–85. doi: 10.1007/s40279-018-1033-y, PMID: 30671905 PMC6445826

[9] Manz F Wentz A. The importance of good hydration for the prevention of chronic diseases. Nutr Rev. (2005) 63:S2–5. doi: 10.1111/j.1753-4887.2005.tb00150.x, PMID: 16028566

[10] Armstrong LE Bergeron MF Muñoz CX Kavouras SA. Low daily water intake profile—is it a contributor to disease? Nutr Health. (2024) 30:435–46. doi: 10.1177/02601060241238826, PMID: 38515347 PMC11402272

[11] Stookey JD Kavarous SA Suh HG Lang F. Underhydration is associated with obesity, chronic diseases, and death within 3 to 6 years in the U.S. population aged 51–70 years. Nutrients. (2020) 12:905. doi: 10.3390/nu12040905, PMID: 32224908 PMC7230456

[12] Kostelnik SB Davy KP Hedrick VE Thomas DT Davy BM. The validity of urine color as a hydration biomarker within the general adult population and athletes: a systematic review. J Am Coll Nutr. (2021) 40:172–9. doi: 10.1080/07315724.2020.1750073, PMID: 32330109

[13] Zhang N Du S Zheng M Tang Z Yan R Zhu Y. Urine color for assessment of dehydration among college men students in Hebei, China – a cross-sectional study. Asia Pac J Clin Nutr. (2017) 26:788–93. doi: 10.6133/apjcn.052017.09 28802286

[14] Baron S Courbebaisse M Lepicard EM Friedlander G. Assessment of hydration status in a large population. Br J Nutr. (2015) 113:147–58. doi: 10.1017/S0007114514003213, PMID: 25418739

[15] Villiger M Stoop R Vetsch T Hohenauer E Pini M Clarys P. Evaluation and review of body fluids saliva, sweat and tear compared to biochemical hydration assessment markers within blood and urine. Eur J Clin Nutr. (2018) 72:69–76. doi: 10.1038/ejcn.2017.136, PMID: 28853743 PMC5765170

[16] Grandjean AC Reimers KJ Buyckx ME. Hydration: issues for the 21st century. Nutr Rev. (2003) 61:261–71. doi: 10.1301/nr.2003.aug.261-271, PMID: 13677588

[17] Tani Y Asakura K Sasaki S Hirota N Notsu A Todoriki H. The influence of season and air temperature on water intake by food groups in a sample of free-living Japanese adults. Eur J Clin Nutr. (2015) 69:907–13. doi: 10.1038/ejcn.2014.290, PMID: 25626408

[18] Ji K Kim Y Choi K. Water intake rate among the general Korean population. Sci Total Environ. (2010) 408:734–9. doi: 10.1016/j.scitotenv.2009.10.076, PMID: 19931118

[19] Malisova O Bountziouka V Panagiotakos DB Zampelas A Kapsokefalou M. Evaluation of seasonality on total water intake, water loss and water balance in the general population in Greece. J Hum Nutr Diet. (2013) 26:90–6. doi: 10.1111/jhn.12077, PMID: 23521514

[20] Malisova O Athanasatou A Pepa A Husemann M Domnik K Braun H. Water intake and hydration indices in healthy European adults: the European hydration research study (EHRS). Nutrients. (2016) 8:204. doi: 10.3390/nu8040204, PMID: 27058557 PMC4848673

[21] McKenzie AL Perrier ET Guelinckx I Kavouras SA Aerni G Lee EC. Relationships between hydration biomarkers and total fluid intake in pregnant and lactating women. Eur J Nutr. (2017) 56:2161–70. doi: 10.1007/s00394-016-1256-3, PMID: 27519184 PMC5579181

[22] Perrier E Rondeau P Poupin M Le Bellego L Armstrong LE Lang F. Relation between urinary hydration biomarkers and total fluid intake in healthy adults. Eur J Clin Nutr. (2013) 67:939–43. doi: 10.1038/ejcn.2013.93, PMID: 23695204 PMC3778844

[23] Zhang J Ma G Du S Zhang N. The relationships between water intake and hydration biomarkers and the applications for assessing adequate total water intake among young adults in Hebei, China. Nutrients. (2021) 13:3805. doi: 10.3390/nu13113805, PMID: 34836061 PMC8623709

[24] Zhang N Du S Tang Z Zheng M Yan R Zhu Y. Hydration, fluid intake, and related urine biomarkers among male college students in Cangzhou, China: a cross-sectional study-applications for assessing fluid intake and adequate water intake. Int J Environ Res Public Health. (2017) 14:513. doi: 10.3390/ijerph14050513, PMID: 28492493 PMC5451964

[25] Zhang J Zhang N Liu S Du S He H Ma G. The comparison of water intake patterns and hydration biomarkers among young adults with different hydration statuses in Hebei, China. Nutr Metab (Lond). (2021) 18:2. doi: 10.1186/s12986-020-00531-2, PMID: 33407667 PMC7789298

[26] Al-Bouwarthan M Quinn MM Kriebel D Wegman DH. A field evaluation of construction workers’ activity, hydration status, and heat strain in the extreme summer heat of Saudi Arabia. Ann Work Expo Health. (2020) 64:522–35. doi: 10.1093/annweh/wxaa029, PMID: 32219304

[27] Piil JF Lundbye-Jensen J Christiansen L Ioannou L Tsoutsoubi L Dallas CN. High prevalence of hypohydration in occupations with heat stress-perspectives for performance in combined cognitive and motor tasks. PLoS One. (2018) 13:e0205321. doi: 10.1371/journal.pone.0205321, PMID: 30356308 PMC6200230

[28] Polat M Akil Í Yuksel H Coskun S Yilmaz D Erguder I. The effect of seasonal changes on blood pressure and urine specific gravity in children living in Mediterranean climate. Med Sci Monit. (2006) 12:CR186–90. PMID: 16572065

[29] Orysiak J Młynarczyk M Tomaszewski P. Hydration status in men working in different thermal environments: a pilot study. Int J Environ Res Public Health. (2022) 19:5627. doi: 10.3390/ijerph19095627, PMID: 35565019 PMC9104106

[30] Mora-Rodriguez R Ortega JF Fernandez-Elias VE Kapsokefalou M Malisova O Athanasatou A. Influence of physical activity and ambient temperature on hydration: the European Hydration Research Study (EHRS). Nutrients. (2016) 8:252. doi: 10.3390/nu8050252, PMID: 27128938 PMC4882665

[31] Tanaka S Fujishiro M Watanabe K Imatake K Suzuki Y Abe M. Seasonal variation in hydration status among community-dwelling elderly in Japan. Geriatr Gerontol Int. (2020) 20:904–10. doi: 10.1111/ggi.14010, PMID: 32827223

[32] Stavrinou PS Giannaki CD Andreou E Aphamis G. Prevalence of hypohydration in adolescents during the school day in Cyprus: seasonal variations. East Mediterr Health J. (2020) 26:1034–41. doi: 10.26719/emhj.20.014, PMID: 33047794

[33] Nakayama A Mitsui T Nakata T Mabuchi H Kawabata K Yoshimatsu H. Changes in thermal comfort, core temperature, and body weight during simulated parcel home-delivery in summer and winter. Ind Health. (2019) 57:604–14. doi: 10.2486/indhealth.2018-0183, PMID: 30713221 PMC6783291

[34] Zhang J Zhang N Liang S Wang Y Liu S Liu S. The amounts and contributions of total drinking fluids and water from food to total water intake of young adults in Baoding, China. Eur J Nutr. (2019) 58:2669–77. doi: 10.1007/s00394-018-1814-y, PMID: 30225629

[35] Ma G Zhang Q Liu A Zuo J Zhang W Zou S. Fluid intake of adults in four Chinese cities. Nutr Rev. (2012) 70:s105–10. doi: 10.1111/j.1753-4887.2012.00520.x, PMID: 23121344

[36] Du S Pan H Hu X Zhang Q Wang X Lu L. Water intake of primary and middle school students in four cities of China. Chin J Prev Med. (2013) 47:210–3. doi: 10.3760/cma.j.issn.0253-9624.2013.03.005, PMID: 23866744

[37] Zhang J Zhang N Wang Y Liang S Liu S Du S. Drinking patterns and hydration biomarkers among young adults with different levels of habitual total drinking fluids intake in Baoding, Hebei Province, China: a cross-sectional study. BMC Public Health. (2020) 20:468. doi: 10.1186/s12889-020-08558-z, PMID: 32268891 PMC7140363

[38] Perrier ET Buendia-Jimenez I Vecchio M Armstrong LE Tack I Klein A. Twenty-four-hour urine osmolality as a physiological index of adequate water intake. Dis Markers. (2015) 2015:231063. doi: 10.1155/2015/231063, PMID: 25866433 PMC4381985

[39] Brinkmann L Gerken M Riek A. Seasonal changes of total body water and water intake in Shetland ponies measured by an isotope dilution technique1. J Anim Sci. (2013) 91:3750–8. doi: 10.2527/jas.2012-5317 23736044

[40] Orysiak J Młynarczyk M Tomaszewski P. Fluid intake at work in foresters working in different thermal conditions. Sci Rep. (2023) 13:15870. doi: 10.1038/s41598-023-41652-x, PMID: 37741879 PMC10518000

[41] Gong W Ma Y Zhang Z Liang J Zhang J Ding G. Validation of 4 estimating methods to evaluate 24-h urinary sodium excretion: summer and winter seasons for college students in China. Nutrients. (2022) 14:2736. doi: 10.3390/nu14132736, PMID: 35807918 PMC9269089

[42] Stuart RO 2nd Hill K Poindexter J Pak CY. Seasonal variations in urinary risk factors among patients with nephrolithiasis. J Lithotr Stone Dis. (1991) 3:18–27. 11536932

[43] Attalla K De S Sarkissian C Monga M. Seasonal variations in urinary calcium, volume, and vitamin D in kidney stone formers. Int Braz J Urol. (2018) 44:947–51. doi: 10.1590/S1677-5538.IBJU.2018.0095, PMID: 29757578 PMC6237522

[44] Yasutake K Nagafuchi M Izu R Kajiyama T Imai K Murata Y. Sodium and potassium urinary excretion levels of preschool children: individual, daily, and seasonal differences. J Clin Hypertens (Greenwich). (2017) 19:577–83. doi: 10.1111/jch.12966, PMID: 28127859 PMC8030865

[45] Lim YH Park MS Kim Y Kim H Hong YC. Effects of cold and hot temperature on dehydration: a mechanism of cardiovascular burden. Int J Biometeorol. (2015) 59:1035–43. doi: 10.1007/s00484-014-0917-2, PMID: 25344017

[46] Enhorning S Melander O Engstrom G Elmstahl S Lind L Nilsson PM. Seasonal variation of vasopressin and its relevance for the winter peak of cardiometabolic disease: a pooled analysis of five cohorts. J Intern Med. (2022) 292:365–76. doi: 10.1111/joim.13489, PMID: 35340071 PMC7613412

[47] Marya RK Sood S Sood AK Maini BK. Seasonal variations in urinary calcium and sodium excretion. Indian J Physiol Pharmacol. (1982) 26:73–6. PMID: 7106963

[48] Perrier E Demazieres A Girard N Pross N Osbild D Metzger D. Circadian variation and responsiveness of hydration biomarkers to changes in daily water intake. Eur J Appl Physiol. (2013) 113:2143–51. doi: 10.1007/s00421-013-2649-0, PMID: 23604869 PMC3714557

[49] Thomas DR Cote TR Lawhorne L Levenson SA Rubenstein LZ Smith DA. Understanding clinical dehydration and its treatment. J Am Med Dir Assoc. (2008) 9:292–301. doi: 10.1016/j.jamda.2008.03.006, PMID: 18519109

[50] Cheuvront SN Ely BR Kenefick RW Sawka MN. Biological variation and diagnostic accuracy of dehydration assessment markers. Am J Clin Nutr. (2010) 92:565–73. doi: 10.3945/ajcn.2010.29490, PMID: 20631205

[51] Versey NG O'Connor H Brotherhood J Graham K. Hydration and its assessment in athletes. Agro Food Ind Hi Tech. (2006) 7:XIV–VII.

[52] Asplund R Aberg H Wetterberg L. The seasonal interrelationship between melatonin, vasopressin, and serum osmolality in elderly subjects. J Pineal Res. (1998) 25:67–72. doi: 10.1111/j.1600-079x.1998.tb00541.x, PMID: 9755026

[53] Shirreffs SM. Markers of hydration status. Eur J Clin Nutr. (2003) 57:S6–9. doi: 10.1038/sj.ejcn.1601895, PMID: 14681707

